# A practical guide for probiotics applied to the case of antibiotic-associated diarrhea in The Netherlands

**DOI:** 10.1186/s12876-018-0831-x

**Published:** 2018-08-06

**Authors:** Valeria Agamennone, Cyrille A. M. Krul, Ger Rijkers, Remco Kort

**Affiliations:** 1 Microbiology and Systems Biology, Netherlands Organization for Applied Scientific Research (TNO), Utrechtseweg 48, 3704 HE Zeist, The Netherlands; 2 University College Roosevelt, Lange Noordstraat 1, 4331 CB Middelburg, The Netherlands; 3 Artis-Micropia, Plantage Kerklaan 38, 1018 CZ Amsterdam, The Netherlands; 4 Department of Molecular Cell Biology, VU University Amsterdam, De Boelelaan 1085, 1081 HV Amsterdam, The Netherlands

**Keywords:** Meta-analysis, Probiotics, Antibiotics, Antibiotic-associated diarrhea (AAD)

## Abstract

**Background:**

Antibiotic-associated diarrhea (AAD) is a side-effect frequently associated with the use of broad spectrum antibiotics. Although a number of clinical studies show that co-administration of specific probiotics reduces the risk for AAD, there is still unclarity among healthcare professionals on the recommendation of probiotic products. This paper aims at a practical guide to inform healthcare professionals, patients and consumers about the exact product characteristics of available probiotics with a proven efficacy to prevent AAD.

**Methods:**

The workflow in this paper includes three consecutive steps: 1) systematic review of relevant clinical studies for effective probiotics by a meta-analysis, 2) compilation of a list of available probiotic products, and 3) recommendation of probiotic products that match effective formulations. Our systematic review on the efficacy of probiotics for the prevention of AAD included only studies with randomized, double blind placebo-controlled trials, a clear definition of antibiotic associated diarrhea, and a probiotic administration regime for at least the duration of the antibiotic therapy.

**Results:**

Using our inclusion criteria, we selected 32 out of 128 identified trials and pooled the results of these studies for each specific dairy product and food supplement. The results indicate a total of seven single or multiple-strain formulations favoring the probiotic treatment group, with the strain *Lactobacillus rhamnosus* GG being the most effective [relative risk ratio of probiotic versus placebo 0.30 (95% CI 0.16–0.5)]. We selected products for recommendation from a compiled list of all probiotic dairy products and food supplements available in The Netherlands and categorized them into groups of products showing effects against the incidence of AAD in at least one, two or three independent clinical studies. We excluded all products which did not unambiguously declare on the label the specific probiotic strain(s) and the number of colony forming units.

**Conclusion:**

Here we present a practical guide that informs healthcare professionals and patients on the availability of probiotic products with a proven efficacy for the prevention of AAD.

**Electronic supplementary material:**

The online version of this article (10.1186/s12876-018-0831-x) contains supplementary material, which is available to authorized users.

## Background

### Antibiotic associated diarrhea (AAD)

**Fig. 1:**
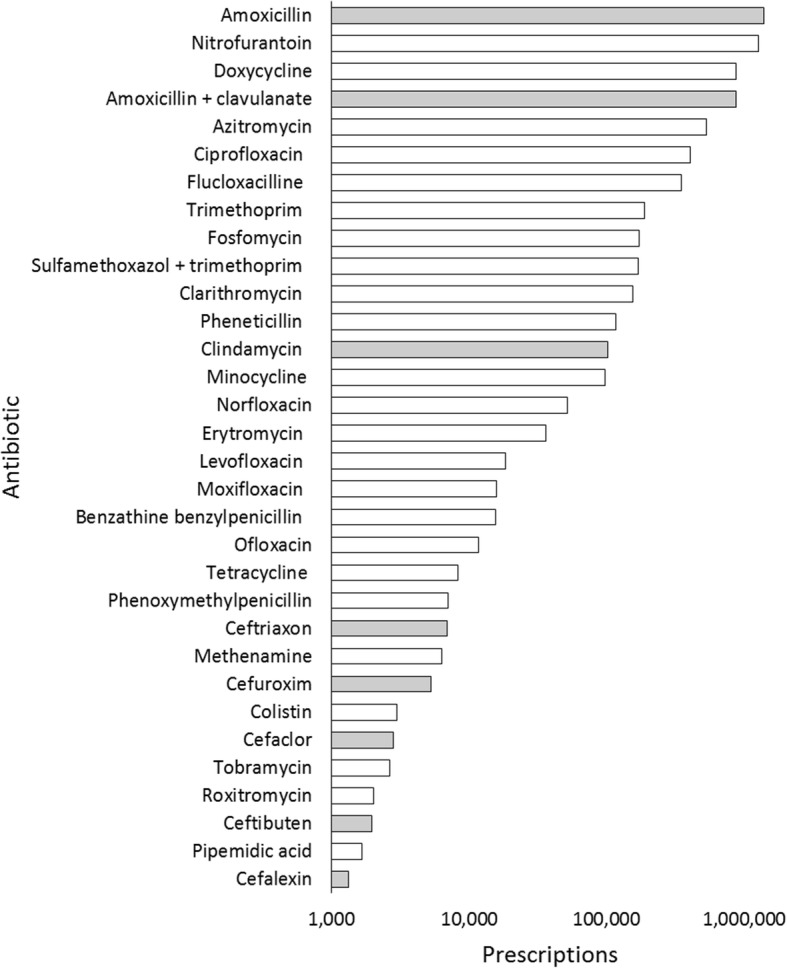
Prescriptions of antibiotics in The Netherlands including those for high risk of AAD. The data have been extracted from the Genees- en hulpmiddelen Informatie Project (GIP; https://www.gipdatabank.nl/databank) from the Zorginstituut Nederland, that collects trends on use of medication in the Netherlands as reported by health insurance companies. Grey bars indicate the antibiotics that are associated with a higher risk of AAD [11, 14–16]

The use of antibiotics is associated with a variety of side-effects. The most common side effects are gastro-intestinal, such as nausea and diarrhea (Additional file 1). Antibiotic-associated diarrhea (AAD) arises when the antibiotic disrupts the ecology of the intestinal microbiota, by altering the diversity and numbers of bacteria in the gut. These changes can affect the capacity of the resident microbiota to resist the invasion of pathogenic microorganisms [1] or the overgrowth of opportunistic pathogens species that are endogenously present in the microbiota [2, 3]. Even after the recovery of total bacterial counts, there can be long-lasting effects on the balance of the intestinal microbiota and consequently on the patient’s susceptibility to infection and other diseases [4, 5]. Therefore, AAD may result in prolonged hospitalization, increased health care costs and other complications. Diarrhea is most frequently associated with the use of broad spectrum antibiotics [6–8], and a tendency to an increase in the prescriptions of broad-spectrum antibiotics has been observed even in a low-prescribing country like the Netherlands (Fig. 1) [9]. For example, the broad-spectrum antibiotic amoxicillin is one of the top 25 drugs that have increased in the numbers of prescriptions in 2015 [10]. Therefore, it is important to consider the methods currently used to contrast the incidence of AAD and to evaluate their efficacy.

### Probiotics as prophylaxis

Probiotics are “live microorganisms that, when administered in adequate amounts, confer a health benefit on the host” [11]. The core benefit of probiotics is exercised by contributing to the maintenance of a balanced microbiota and therefore by creating a favorable gut environment [12]. Furthermore, probiotics support the health of the digestive tract and the immune system [12]. The positive effect of probiotics on gut health in a variety of conditions (antibiotic-associated and infectious diarrhea, irritable bowel syndrome, necrotizing enterocolitis, etc.) has been evaluated by a number of randomized controlled clinical trials [13]. Probiotics can antagonize pathogenic microorganisms in a variety of ways. They can compete with pathogens for nutrients and adhesion sites on the gastrointestinal mucosa [14, 15] in the process of competitive exclusion [16]. They can also prevent pathogenicity by interfering with signaling between pathogens by degrading quorum sensing molecules [17]. In addition, direct antagonism can occur through the production of bacteriocins or metabolites with antimicrobial activity against pathogenic microorganisms [18, 19]. Finally, probiotics are able to modulate and stimulate local and systemic immune responses in the patient [20].

According to the Agency for Healthcare Research and Quality (AHRQ) there is not enough information to confidently judge the safety of probiotic-based interventions [21]. This is because many clinical trials do not adequately document adverse events, and also because rare adverse events are difficult to assess. Still, probiotic products are generally regarded as safe, and they are used both by healthy and ill people globally. Possible safety concerns include diseases such as bacteremia and fungemia [22], and are especially concerning for patients with a weakened or compromised immune system (critically ill infants, post-surgery and hospitalized patients, immuno-compromised patients are at high risk [23, 24]). Additionally, probiotics can constitute a source of antibiotic resistance genes. Although commercial probiotic strain are tested for the presence of such genes, reports have documented the presence of antibiotic resistance in probiotic bacteria from dietary supplements [25, 26].

### Scope of the paper

In this paper we present a practical guide to the use of probiotics for the prevention of antibiotic-associated diarrhea. The guide is based on available scientific evidence and developed by following a workflow in three steps: 1) evaluation of the efficacy of probiotics in the context of AAD and identification of effective strains/formulations by a systematic review of relevant clinical trials and meta-analysis of their results; 2) identification of probiotic products available to the target population; 3) recommendation of specific probiotic products matching effective formulations. The scope of this guide is to inform healthcare professionals and patients on the availability of probiotic products with a proven efficacy for the prevention of AAD.

## Methods

### Search strategy and inclusion criteria

Included in this review are studies that assessed the efficacy of probiotics in reducing the incidence of antibiotic-associated diarrhea (AAD) in patients treated with antibiotics, regardless of their age, of the intervention setting (hospital or outpatients) and of the indication for which they were prescribed. In order to identify these studies we first screened the references listed by previously published systematic reviews and meta-analyses, and then we directly searched clinical trials in public databases. Database searches were conducted on the 16th of January 2017.

We searched reviews and meta-analyses on the following databases for the period 1960–2016: the Database of Abstracts of Reviews of Effects (DARE), the Cochrane Database of Systematic Reviews (CDSR) and PubMed. For the DARE and the CDSR databases we searched combinations of the following terms: “probiotic”, “antibiotic”, “diarrhea” and “antibiotic-associated diarrhea”. The search yielded 25 results from the DARE and 50 from the CDSR. In PubMed, we searched for meta-analyses using the following search texts: “((probiotic[Title/Abstract] OR probiotics[Title/Abstract]) AND (antibiotic associated diarrhea[Title/Abstract] OR antibiotic-associated diarrhea[Title/Abstract] OR antibiotic associated diarrhoea[Title/Abstract] OR antibiotic-associated diarrhoea[Title/Abstract]))”, which yielded 28 results; and “((diarrhea[Title/Abstract] OR diarrhoea[Title/Abstract]) AND (probiotic[Title/Abstract] OR probiotics[Title/Abstract]) AND (antibiotic[Title/Abstract] OR antibiotics[Title/Abstract]))”, which yielded 34 results. After screening of titles and abstracts and exclusion of duplicates and reviews not relevant to our purpose, we identified 28 relevant reviews and meta-analyses [13, 27–53], containing a total of 102 relevant studies.

To confirm and update the information obtained from previously published reviews, we also directly searched clinical trials on the Cochrane Central Register of Controlled Trials (CENTRAL), PUBMED and Excerpta Medica Database (EMBASE) for the period 2010–2017. We used the following search texts: “probiotic AND antibiotic associated diarrhea OR antibiotic associated diarrhoea” and “probiotic AND antibiotic AND diarrhea”. These searches yielded 26 studies.

Among the studies resulting from our searches, we defined the relevant ones on the basis of specific inclusion criteria: (i) randomized trial, with a double-blind setup and including a placebo control, (ii) clear definition of AAD, and incidence of AAD measured as one of the outcomes, and (iii) probiotic administered for at least the duration of antibiotic therapy. Studies that did not meet the above inclusion criteria were excluded. Furthermore, we excluded studies when diarrhea was already present at the start of the intervention and when the probiotics were tested in combination with other products. We also excluded studies not written in English, studies that were not published or not available, and duplicates (studies reporting results already included in another publication). One author screened the abstract and the body of the papers and extracted information relevant to establish eligibility and conduct the meta-analysis. When the full text of a publication was not accessible, relevant information was obtained from previous reviews. The study was only included when the information available from the other sources met the inclusion criteria.

### Data analysis

For each of the included trials, we calculated the relative risk (RR) and the 95% confidence interval (CI) for the incidence of diarrhea in the probiotic versus placebo treatment. In addition, we conducted a subgroup analysis by pooling studies based on the composition of the probiotic Microsoft Excel (2016); the Meta-Essentials tool was used to measure heterogeneity and risk of bias [54].

### Available probiotic products in the Netherlands

A complete list of probiotic products available in The Netherlands was obtained in December 2016 by screening online websites of pharmacies, vitamin stores, health stores, and shops selling probiotics online. The Dutch association Natuur- en Gezondheidsproducten Nederland (NPN, Amersfoort, The Netherlands) evaluated and completed our list. We also included dairy products routinely sold in food stores.

### Recommendations

Based on the effectiveness of probiotic strains included in this review in preventing AAD and on the number of studies supporting it, we defined three categories of recommendations. The categories include (i) a three-star recommendation for significant effects for the reduction of AAD shown in at least three of our selected studies, (ii) a two-star recommendation for effects shown in at least two of our selected studies, and (iii) a one-star recommendation for an effect shown in only one study, a trend supported by two or more studies, or the presence of a strain that satisfies one of the above criteria (showing an effect in one study or a trend in at least 2 studies) in sufficient amounts in food supplement or dairy product with a mixed formulation. We screened the list of products available in the Netherlands and selected products that satisfied the criteria above and that contained the relevant strain(s) at a daily dose at least equal to the lowest dose showing an effect in the included studies. For dairy products, we only recommended those that had been specifically included in a clinical trial.

## Results

### Search strategy and study selection

**Fig. 2:**
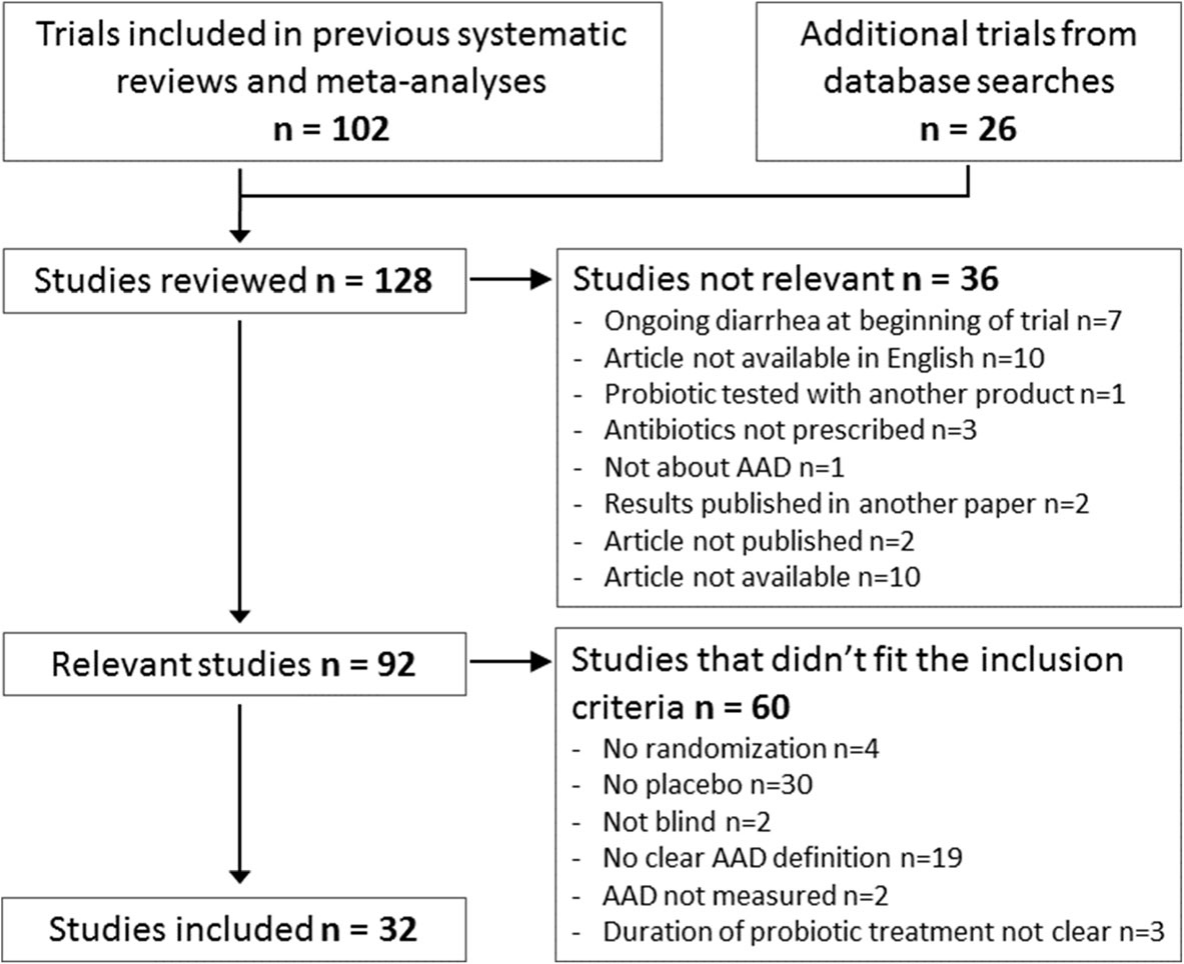
Flow diagram of study selection

The flow of the meta-analysis, from search to study selection, is depicted in Fig. 2. The literature search identified 128 relevant studies. An overview of these studies, including the reasons for their exclusion, is presented in Additional file 2. A total of 32 trials satisfied our inclusion criteria and were included in the meta-analysis: 26 were obtained from previous reviews [55–80] and six from direct database searches [81–86]. The specific characteristics of all included studies are summarized in Additional file 3.

**Fig. 3:**
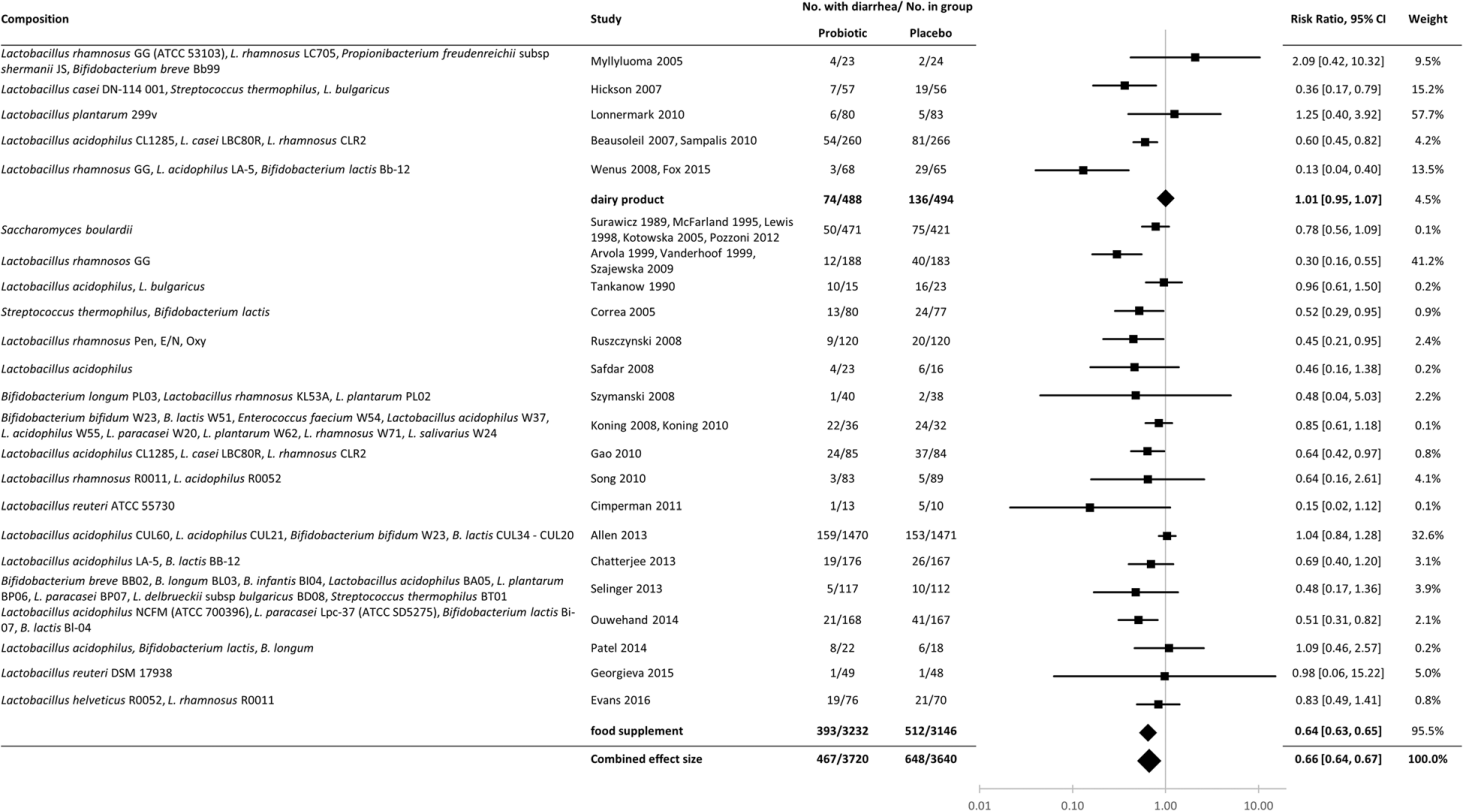
Forest plots of the subgroup meta-analysis of probiotics for AAD (food supplements vs dairy products). Studies are pooled based on composition. Note that we pool all the studies on *Saccharomyces boulardii* as we consider this to be one strain of *Saccharomyces cerevisiae* [93]

Most of the studies that were identified through our search, but were excluded from the meta-analysis, did not include a placebo (31%). The second largest fraction of excluded studies consisted of studies without a clear definition of AAD (20%). Among other reasons for exclusion were the lack of a precise measurement of diarrhea, and an unclear duration of the probiotic treatment.

From the total of 32 included studies, five (15.6%) did not specify the antibiotic used in the trial. Six studies (9%) used only one antibiotic or combination of antibiotics: two studies used amoxicillin, one used amoxicillin with clavulanic acid, one used a non-specified beta-lactam antibiotic, and two used a combination of three different antibiotics to eradicate *Helicobacter pylori* infection. The remaining 21 studies (60%) enrolled patients that were taking different antibiotics. All of these studies included antibiotics associated with a high risk of AAD, including amoxicillin, beta-lactams, broad-spectrum penicillins, cephalosporins and clindamycin. Ten studies focused on children up to 17 years old (of which one focused on infants 6–36 months), and 22 on adults (three of which focused on elderly over 65 years old).

### Data analysis

The results of the meta-analysis have been summarized in Fig. 3. In this forest plot, studies are pooled based on probiotic formulation and sub-grouped in two categories: dairy products (7 studies) and food supplements (i.e. non-dairy products, 25 studies). Results are also reported for each individual trial in chronological order of publication date in Additional file 4. Overall, probiotics were associated with lower incidence of antibiotic-associated diarrhea (467/3720 [13%]) compared to the control (648/3640 [18%]) (RR 0.66, 95% CI 0.64–0.67). For trials using probiotic dairy products, the incidence of AAD in the probiotic group was 15.2% compared to 27.5% in the control group (RR 1.01, 95% CI 0.95–1.07). For trials including food supplements (i.e. non-dairy products) the incidence of diarrhea in the probiotic group was 12.2% compared to 16.3% in the control group (RR 0.64, 95% CI 0.63–0.65).

### Recommendations

A compiled list of probiotics available in The Netherlands in December 2016 is presented in Additional file 5. We identified the following strains satisfying the recommendation criteria: *Lactobacillus rhamnosus* GG with a minimal daily dose of 2 × 10^9^ CFU, to which we assigned a three-star recommendation, as it is associated with a significant reduction in the incidence of AAD in at least three of our selected studies[55, 76, 79]. In addition, the multi-strain formulation of *Lactobacillus rhamnosus* GG, *Lactobacillus acidophilus* LA-5, *Bifidobacterium lactis* BB-12 shows a significant effect in the reduction of AAD in two of our selected studies, but we did not identify an available probiotic product containing this formulation (see further below) [59, 80]. A number of multi-strain formulations led to a one-star recommendation, including those significantly reducing the incidence of AAD in only one selected study: *Streptococcus thermophilus* and *Bifidobacterium lactis* (minimal daily dose: 5 billion CFU) [58], *Lactobacillus rhamnosus* strains Pen, E/N, Oxy (min daily dose: 4 billion CFU) [71] and *Lactobacillus acidophilus* CL1285, *Lactobacillus casei* (minimal daily dose: 50 billion CFU) [60]. Furthermore, we assigned a one-star recommendation to formulations that showed a trend supported by two or more studies, including *Saccharomyces boulardii* (minimal daily dose: 10 billion CFU) [65, 66, 68, 70, 75] and *Bifidobacterium bifidum*, *Bifidobacterium lactis*, *Enterococcus faecium*, *Lactobacillus acidophilus*, *Lactobacillus paracasei*, *Lactobacillus plantarum*, *Lactobacillus rhamnosus*, *Lactobacillus salivarium* (minimal daily dose: 10 billion CFU) [63, 64].

We then identified probiotic products (both food supplements and dairy products) to recommend based on the categories above. We recommend food supplements and dairy products that showed a significant effect favoring the probiotic in at least three independent clinical trials in our meta-analysis. No dairy product showed a significant effect for the reduction of the incidence of AAD in at least three clinical studies, and the only food supplement that shows such an effect in at least three clinical studies is *Lactobacillus rhamnosus* LGG (minimal daily dose: 2 billion CFU) [46, 55, 79]. If we consider the products available in the Netherlands, the recommended products are the food supplements Microbiol Platinum (Vitals) and Culturelle (Allergy Research Group) containing *Lactobacillus rhamnosus* GG at a daily dose of 33 and 10 billion CFU, respectively (Table 1). We did not identify any dairy products of food supplements products in our two-star category. For the one star category we identified one dairy product and five food supplements containing either the exact (combination of) probiotic(s) that showed a significant effect against AAD in one clinical trial or a trend in at least two clinical trials, or combinations of different probiotic bacteria, for some of which this effect or trend was proven. We only list and recommend products for which the probiotic strain(s) and CFU-counts are specified and correspond to the strain and dose showing an effect or trend favoring the treatment in the included studies. The one-star dairy products is Actimel (Danone) (daily dose of 20 billion CFU) [62] and the food supplements include Probioticum (Wapiti), Winbiotic Pro-AD (Winclove), Probactiol Duo (Metagenics), Advanced Multi-Billion Dophilus (Solgar), and Imutis (Trenker), as listed in Table 1.

**Table 1:** List of recommended probiotic products

Category	Brand name	Manufacturer	Probiotic strain	CFU per dose	Total daily dose
Three-star	Microbiol Platinum	Vitals	*Lactobacillus rhamnosus* GG	3.3 × 10^10^	1 capsule
Three-star	Culturelle	Allergy Research Group	*Lactobacillus rhamnosus* GG	1.0 × 10^10^	1 capsule
One-star	Actimel (dairy product)	Danone	*Lactobacillus casei* DN-114001	1.0 × 10^10^	1 bottle (100 ml)
One-star	Probioticum	Wapiti	*Saccharomyces boulardii*	2.5 × 10^9^	1–4 capsules
One-star	Winbiotic Pro-AD	Winclove	*Bifidobacterium bifidum* W23	1.1 × 10^9^	2 sachets
			*Bifidobacterium lactis* W51	1.1 × 10^9^	
			*Enterococcus faecium* W54	1.1 × 10^9^	
			*Lactobacillus acidophilus* W37	1.1 × 10^9^	
			*Lactobacillus acidophilus* W55	1.1 × 10^9^	
			*Lactobacillus paracasei* W20	1.1 × 10^9^	
			*Lactobacillus plantarum* W62	1.1 × 10^9^	
			*Lactobacillus rhamnosus* W71	1.1 × 10^9^	
			*Lactobacillus salivarius* W24	1.1 × 10^9^	
One-star	Probactiol Duo	Metagenics	*Saccharomyces boulardii*	6.0 × 10^9^	1–2 capsules
			*Lactobacillus acidophilus* NCFM	2.1 × 10^9^	
			*Lactobacillus paracasei* Lpc-37	2.1 × 10^9^	
			*Bifidobacterium lactis* Bi-04	2.1 × 10^9^	
			*Bifidobacterium lactis* Bi-07	2.1 × 10^9^	
One-star	Imutis	Trenker	*Saccharomyces boulardii*	6.0 × 10^9^	1–4 capsules
			*Lactobacillus acidophilus*	2.0 × 10^9^	
			*Lactobacillus rhamnosus*	3.0 × 10^9^	
			*Bifidobacterium longum*	2.0 × 10^9^	
One-star	Advanced Multi-Billion Dophilus	Solgar	*Lactobacillus acidophilus* LA-5	1.3 × 10^9^	1 capsule
			*Lactobacillus paracasei* L CASEI 431	1.3 × 10^9^	
			*Lactobacillus rhamnosus* GG	1.3 × 10^9^	
			*Bifidobacterium lactis* BB-12	1.3 × 10^9^	

## Discussion

### Study selection and inclusion criteria

In this review we chose to only include studies that had a clear definition of antibiotic-associated diarrhea, to be able to compare their results in a systematic way. However, studies lacking a precise definition of diarrhea may still provide valuable information, and it could be a subject for future discussions how to interpret them and whether to take them into account when formulating recommendations. Furthermore, the strict definition of diarrhea used in some studies means that the protective effect of probiotics against AAD may have been underestimated [67]. Given the scope of the review, we searched for clinical trials involving the use of antibiotics, but we didn’t apply strict inclusion criteria regarding the kind of antibiotic used. We didn’t look for studies using specific treatments, nor did we exclude studies that did not indicate which antibiotics they used, since diarrhea can be a side-effect of many. Five of the studies that we included did not specify which antibiotic was administered to the patients during the clinical trial. Of the remaining 27 studies, 21 enrolled patients taking different antibiotics, including antibiotic such as broad-spectrum penicillins and cephalosporins associated with a high-risk of AAD.

Only some studies reported which antibiotics were used among the characteristic of patients following treatment [58, 65, 71]. Kotowska et al. [65] suggested that the probiotic they tested (*Saccharomyces boulardii*) may be effective in preventing diarrhea caused by amoxicillin with clavulanate and by intravenous ceforuxime, but they also mentioned that they could not make definitive conclusions regarding differences in the probiotic’s efficacy against different classes of antibiotics. Similarly, other studies could not detect significant differences in this regard, either because of a small sample size (relative to the number of antibiotics tested) or because of the low incidence of diarrhea in the study, or both [75, 82]. Twenty-two of the studies included in this review included a power analysis. Of these, 11 detected a significant difference between treatment and placebo, including two studies that were underpowered according to their power analysis [56, 65]. Of the 11 studies that did not detect a significant difference between the treatment and the placebo, four were underpowered [72–74, 85]. By ensuring that clinical trials have enough power it would be possible to identify which probiotics are most effective in preventing diarrhea caused by specific antibiotics, and clinicians would be able to recommend different probiotic products based on the antibiotic therapy prescribed to their patients.

An important aspect in the design of clinical trials is the inclusion of a placebo control group. This kind of control allows clinicians to account for the placebo effect, which is a well-recognized phenomenon in clinical practice. In clinical trials with dairy products the placebo would ideally consist of a specifically developed product with organoleptic properties very similar to the dairy product containing the probiotics. However, in case of the clinical trials testing probiotic dairy drinks included in this review, the placebo is often a different product. Since we are not aware to which extent these product differences affect the placebo response in individual patients, it may be opportune for producers of probiotic dairy drinks to develop products that can be administered as more appropriate placebos in clinical trials.

### Criteria for recommendations

In this review, we adopted strict criteria to derive recommendations from the results of our meta-analysis. Specifically, we decided to limit strong recommendations for commercial products for which the specific probiotic combination was tested, and not the single species separately, and for which the efficacy of the composition is supported by at least three clinical trials. This approach, although necessary to ensure evidence-based decision making, is limiting, since there are likely other products on the market whose exact composition has not been tested but that may be effective in preventing AAD. In fact, many of the works that we reviewed that assessed the efficacy of multi-strain probiotics (more than three strains) in reducing the risk of AAD, concluded that these products had a significant effect on risk reduction [63, 64, 86].

For dairy products, we recommended those that were shown to have a positive effect in a clinical trial, but it is possible that products from other brands, with a similar formulation, may be as effective as those tested. For example, we reviewed here a study showing a positive effect of the probiotic dairy drink Actimel (Danone) in preventing diarrhea caused by antibiotics [62], and we subsequently included this product in the list of one-star recommendations. The brand Yakult produces a dairy drink containing a strain of *Lactobacillus casei* that has been shown to be virtually identical to the strain used by the brand Actimel [87], providing an argument for a one-star recommendation to the Yakult dairy drink without the need to conduct additional clinical trials.

Although our recommendations are based on different criteria and are not limited to children, they are in line with those of the European Society for Pediatric Gastroenterology, Hepatology and Nutrition (ESPGHAN) working group [53]. The strain *L. rhamnosus* GG, for which we make a three-star recommendation, was also strongly recommended for the prevention of AAD in children by the ESPGHAN working group, on the basis of a moderate quality of evidence. The working group also gave a strong recommendation to *S. boulardii*. However, we could not do the same on the basis of our analysis, because we pooled results of patients of different ages. In the forest plot in Additional file 4, *S. boulardii* shows a positive effect in the prevention of AAD in children [65], a positive trend in adults [68, 75], but no positive effect in elderly [66, 70]. Age is one of the factors that should be taken into account when evaluating health benefits of probiotics. In general, differences in the inclusion criteria, in the methods used to conduct the meta-analysis and in the criteria used to formulate recommendations will result in different evidence-based advice.

### Factors affecting the efficacy of probiotics

Multiple factors can determine the efficacy of probiotic products in specific therapeutic contexts. Firstly, the efficacy of a product can be influenced by its strain composition. One of the most studied probiotic strains is *Lactobacillus rhamnosus* GG, which has been repeatedly proven effective in reducing in the incidence of diarrhea in antibiotic-treated patients and in treating other gastrointestinal disorders [88]. Different strains of *L. rhamnosus* may not be equally effective in preventing the incidence of side effects of antibiotics [13], and the same is true for other probiotic species. Clinical trials should always specify which probiotic strain they tested, however this is not always the case, making it difficult to evaluate and compare their results. Furthermore, genetic variability has been observed among “identical” strains of LGG [89], so even when studies indicate precisely which strain they used it is not possible to exclude the possibility of within-strain differences affecting the results of the trial. Apart from strain composition, the formulation of a probiotic product (specific combination of strains) may affect its efficacy. This effect may be particularly significant in dairy products, since the quality of the product will vary depending on the specific strains used during the fermentation, and whether they are included during the process or added as ingredients to the final product. In this review we have analyzed dairy products and food supplements separately, and we have only combined probiotic products with the exact same strain composition and formulation, in order to minimize the effect of these factors on the results of the meta-analysis.

Apart from strain composition and probiotic product formulation, specific individual differences (age, specific health condition, genetic factors and differences in the composition of the gut microbiome) might play a role in the efficacy of probiotics, as is evident in some of the trials we reviewed.

The largest study included in this review contained almost 3000 subjects, as reported by Allen [81]. This study showed no significant effect of probiotic versus placebo. However, it included elderly participants (over 65) who may be more susceptible to adverse effects of antibiotics.. The efficacy of probiotics varies across different age groups, and is influenced by the type of antibiotic administered and the duration of the therapy. In fact, higher incidence rates of AAD were previously observed in older patients also subjected to prolonged antibiotic exposure [84], so the same factors may partly explain the observation of the study by Allen. Furthermore, in the study by Allen antibiotic therapy could last up to 7 days before starting the probiotic treatment, and probiotics may be more effective when administered during the entire period of susceptibility. In fact, a meta-regression analysis conducted by Shen et al. [90] showed that probiotics were significantly more effective in reducing the risk of *Clostridium difficile* infection when administered closer to the first antibiotic dose, and similar considerations could be applied to the use of probiotics to prevent AAD.

The efficacy of probiotics in preventing AAD also depends on the dose. A daily intake of at least 5 × 10^9^ CFU is associated with significant efficacy for AAD [41, 91], and it has been shown that higher probiotic dose is linked to greater efficacy [60, 84]. Although only few dose-effect studies have been performed, they observe a positive correlation between dose and AAD risk [92].

Since so many factors can affect the efficacy of probiotics in prophylaxis, researchers should be rigorous in setting up clinical trials and in providing as much information as possible about them. Studies should report characteristics of the probiotic (strain, dose and duration of therapy), of the antibiotic (type of antibiotic, duration of the therapy) of the patients (age group, diagnosis) and accurate definitions of measured outcomes and adverse effects. In this way, results from different trials can be assessed, compared and used as a basis to formulate recommendations. Individual factors, that are not routinely monitored in clinical trials, may influence the incidence and gravity of side effects and the efficacy of probiotics. For example, each individual has their own unique microbiota, and the impact of a given antibiotic on the composition and stability of different microbial ecosystems can be different; therefore, a specific probiotic strain or combination of strains may not have the same efficacy for every person. Especially for some patients, for example those who are frequently treated with antibiotics such as elderly in care facilities, it is certainly worth being flexible and trying different probiotics until the most effective one has been found. Future research can guide the formulation of personalized therapies.

## Conclusion

We present here a workflow for the assessment of the efficacy of probiotics for the prevention of antibiotic-associated diarrhea. The workflow consists of a series of steps (systematic review of available literature and meta-analysis of relevant clinical trials, inventory of available products and formulation of evidence-based recommendations) that can be applied to other cases, upon adaptation of methodological details such as the inclusion criteria. In order to make strong, evidence-based recommendations it is important that research of high-quality is available, in which adequate methods are followed to perform the trials and to report the results. We conclude that there is sufficient evidence to make a recommendation for the use of specific probiotic products for the prevention of antibiotic associated diarrhea. In particular, we provide a three-star recommendation for preparations with a minimal daily dose of 2 × 10^9^ CFU of the probiotic strain *Lactobacillus rhamnosus* GG.

## Additional files

Additional file 1: Side-effects of antibiotics. List of commonly prescribed antibiotics in The Netherlands, with information on class, mode of action, indication and side effects. (XLSX 29 kb)

Additional file 2: List of reviewed studies. All the clinical trials that were reviewed are listed with indication of first author, publication title, publication date, whether they were included in the review, and, in case, reasons for exclusion. (XLSX 26 kb)

Additional file 3: Characteristics of included studies. For every study included in this review, information on study population (number of patients enrolled, age, setting, country, diagnosis and antibiotics prescribed), probiotic treatment (composition, daily dose, intake form, brand, duration of treatment) and outcome (definition of AAD, incidence in probiotic and placebo groups, significance of difference, relative risk) is summarized. (XLSX 39 kb)

Additional file 4: Probiotics for prevention of antibiotic-associated diarrhea (AAD) in 32 randomized, double-blind, placebo-controlled trials. Forest plot summarizing the results of the meta-analysis for all the clinical trials included in this review, listed in chronological order of publication date. (PNG 83 kb)

Additional file 5: Probiotic products available in The Netherlands. List of probiotic products available in the Netherlands, with information on composition (strains), dosage form, and number of colony-forming units (CFU) per daily dose. (XLSX 54 kb)

## Abbreviations

AAD: Antibiotic-associated diarrhea
AHRQ: Agency for Healthcare Research and Quality
CDSR: Cochrane Database of Systematic Reviews
CENTRAL: Cochrane Central Register of Controlled Trials
CFU: Colony-forming units
CI: Confidence interval
DARE: Database of Abstracts of Reviews of Effects
EMBASE: Excerpta Medica Database
ESPGHAN: European Society for Pediatric Gastroenterology, Hepatology and Nutrition
NPN: Natuur- en Gezondheidsproducten Nederland
RCT: Randomized controlled trial
RR: Relative risk
