# Supplementary figures and images for: A practical guide for probiotics applied to the case of antibiotic-associated diarrhea in The Netherlands
Source: BMC Gastroenterol. 2018 Aug 6;18:103. doi: 10.1186/s12876-018-0831-x (PMC6091175; doi:10.1186/s12876-018-0831-x)

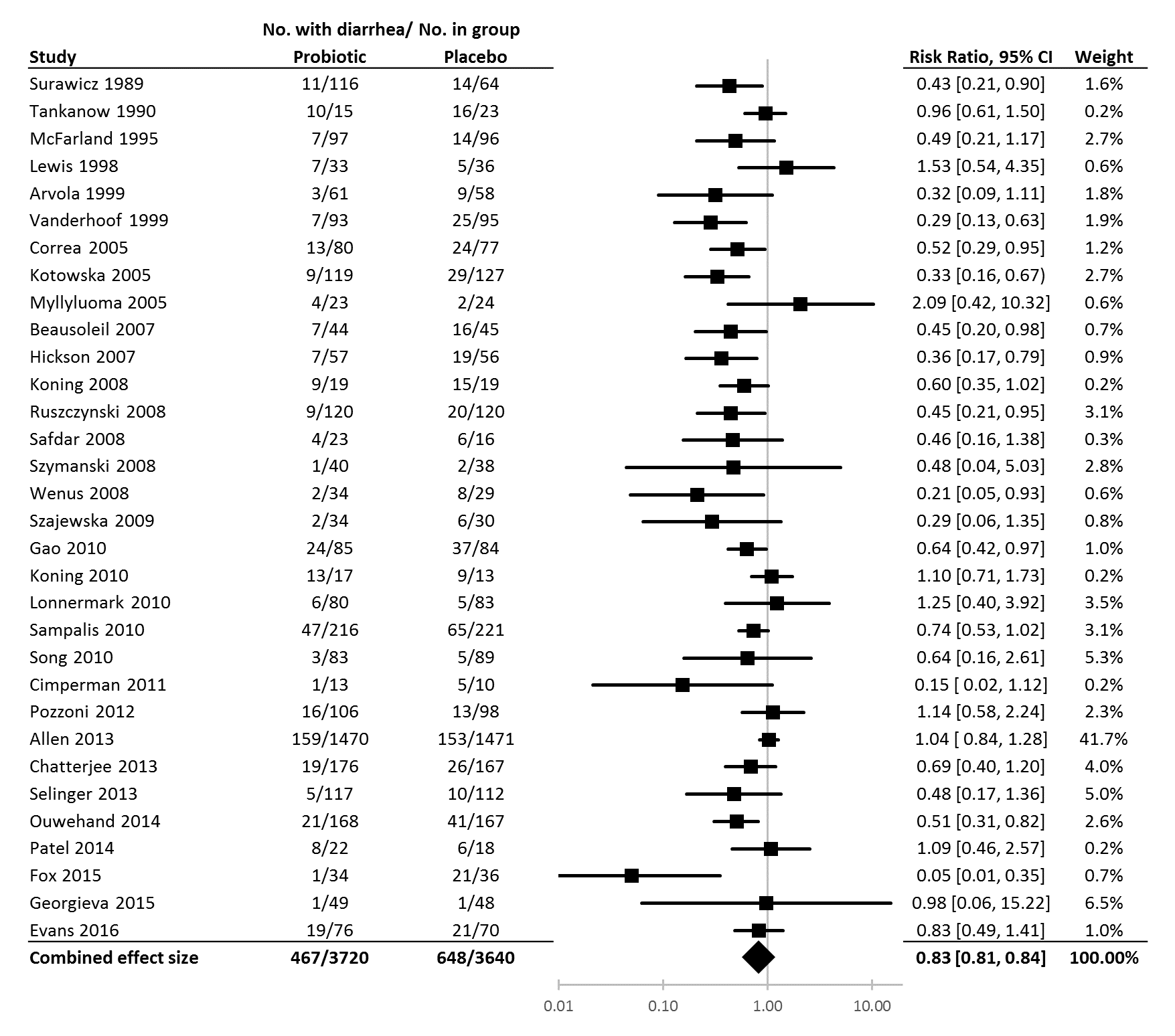

Supplement: Supplementary file 4 — Probiotics for prevention of antibiotic-associated diarrhea (AAD) in 32 randomized, double-blind, placebo-controlled trials. Forest plot summarizing the results of the meta-analysis for all the clinical trials included in this review, listed in chronological order of publication date. (PNG 83 kb) [file 12876_2018_831_MOESM4_ESM.png]
